# New Simplified White Blood Cells Score Improves Mortality Prediction in Severe COVID-19 Patients

**DOI:** 10.3390/jcm15072590

**Published:** 2026-03-28

**Authors:** Kamil Paryż, Arkadiusz Lubas, Mateusz Gutowski, Bartosz Rustecki, Andrzej Michałowski, Jakub Klimkiewicz

**Affiliations:** 1 Department of Anesthesiology and Intensive Care, Military Institute of Medicine—National Research Institute, Szaserów 128 Str., 04-141 Warsaw, Poland; 2 Department of Nephrology, Internal Diseases and Dialysis, Military Institute of Medicine—National Research Institute, Szaserów 128 Str., 04-141 Warsaw, Poland

**Keywords:** COVID-19, WBCS, white blood cells score, mortality, neutrophil, lymphocyte, eosinophil

## Abstract

**Background**: An unfavorable course of SARS-CoV-2 infection can lead to significant morbidity and mortality. The study aimed to develop a simple, accessible, and reliable tool to anticipate the poor results among COVID-19 pneumonia patients. **Methods**: This retrospective cohort study involves 306 individuals with severe COVID-19 pneumonia enrolled between March 2021 and June 2021. Each patient had confirmed SARS-CoV-2 infection and required oxygen therapy. Differential blood count and serum CRP were taken on admission day. Medical data were collected from the hospital’s information system. **Results**: Of 306 patients (133 females, 173 males, aged 66.3 ± 15.2 years), 105 (34.3%) died. Counts of neutrophils, lymphocytes, and eosinophils differed significantly between survivors and deceased (*p* < 0.001; *p* = 0.002; *p* = 0.009, respectively) and had substantially differentiating properties in ROC analysis. Built with the counts of neutrophils, lymphocytes, and eosinophils, the White Blood Cell Score (WBCS) was developed. WBCS robustly predicted mortality (OR = 2.821; CI: 2.037–3.906; *p* < 0.001) in the investigated population. Cumulative risk of death according to WBCS (ranging from 0 to 3 points) was as follows: 0 points—10.9%, 1 point—23.5%, 2 points—33.1%, 3 points—34.1%. **Conclusions**: Based on differential blood count, the proposed WBCS is easy to use and can be helpful in predicting mortality among severe COVID-19 patients.

## 1. Introduction

COVID-19 is a viral disease caused by severe acute respiratory syndrome coronavirus 2 (SARS-CoV-2), primarily affecting the respiratory system. Severe course of COVID-19 is accompanied by systemic inflammatory response syndrome (SIRS), which—in many cases—leads to multiple organ dysfunction syndrome (MODS) [1,2,3]. According to the WHO, by January 2026, 779,102,516 COVID-19 cases were reported worldwide, of which 7,110,188 were fatal (0.9%) [4]. Several comorbidity-related risk factors were identified, such as cardiovascular diseases, chronic respiratory system diseases, obesity, chronic kidney disease, diabetes, cerebrovascular diseases, and neoplasm [2,5,6].

In this paper, we use the WHO definitions of disease severity for COVID-19 for adults:

- Critical COVID-19—Defined by the criteria for acute respiratory distress syndrome (ARDS), sepsis, septic shock, or other conditions that would normally require the provision of life-sustaining therapies such as mechanical ventilation (invasive or non-invasive) or vasopressor therapy;
- Severe COVID-19—Defined by oxygen saturation <90% on room air; severe pneumonia; signs of severe respiratory distress (in adults, accessory muscle use, inability to complete full sentences, respiratory rate >30 breaths per minute);
- Non-severe COVID-19—Defined as the absence of any criteria for severe or critical COVID-19 [7].

Several studies have shown a significant correlation between the severity of the COVID-19 course and several selected biomarkers, such as interleukin 6, neutrophil to lymphocyte ratio (NLR), C-reactive protein (CRP), ferritin, LDH, d-dimers, and troponin I [3,5,8,9,10,11,12,13,14]. Coagulopathy is another high-risk factor for morbidity and mortality in the COVID-19 course [15]. CRP and NLR are of most interest during a shortage of staff and high load of severe COVID-19 cases due to their accessibility and low costs.

NLR is calculated with a complete blood count (CBC). It is obtained by dividing the neutrophil count by the lymphocyte count. NLR combines the two immunological responses—innate, non-specific, represented by neutrophil count, and humoral, specific, measured with lymphocyte count. NLR value can be elevated in many clinical scenarios involving tissue damage and generalized inflammation. The most common are infections—typical bacterial or fungal, traumas, myocardial infarction, cerebral ischemic stroke, neoplasm, and post-operative complications [16]. Studies have shown a correlation between elevated NLR and mortality risk in the general population [16,17]. Moreover, higher NLR has been linked with poor prognosis in patients with sepsis, community-acquired pneumonia (CAP), and COVID-19 pneumonia, and also NLR has been considered as a predictor of cardiovascular events and post-operative complications [16]. Despite the evidence that NLR is an independent predictor of mortality in severe diseases, the cut-off values for poor prognosis are still discussed [16]. Several factors can affect NLR values and falsely elevate them, e.g., steroids or granulocyte colony-stimulating factor (G-CSF) administration, hematologic diseases, chemotherapy, HIV infection, age, and obesity [3,5,11,12,16].

C- reactive protein (CRP) is an acute-phase protein, which is a non-specific indicator of inflammatory response. It can be elevated in many situations, such as infection, trauma (including elective surgical procedure), myocardial infarction, pancreatitis, and autoimmune diseases [18]. What is more, obesity, pregnancy, depression, diabetes, and smoking could result in a slight elevation of CRP concentration. Studies have shown that in COVID-19, elevated CRP concentration is associated with a higher risk of developing pneumonia and severe respiratory failure [9,18,19,20].

COVID-19 has a continuum of clinical manifestations, varying from asymptomatic, through respiratory failure, up to multiorgan failure [1,3]. Thus, there is a high demand for developing clinical tools to identify individuals at risk for disease progression.

Taking the above into consideration, we decided to perform this retrospective cohort study, aiming to develop a clinical tool to anticipate the severe course of COVID-19. We decided to focus on using CBC and serum CRP, as they are widely accessible and often used in clinical practice.

## 2. Materials and Methods

### 2.1. Patients

This retrospective study enrolled 306 patients with severe COVID-19 admitted to the COVID-19 Hospital of the Military Institute of Medicine in Warsaw, Poland, between March 2021 and June 2021. Only individuals with SARS-CoV-2 infection confirmed with a polymerase chain reaction test (GeneFinder COVID-19 Plus RealAmp Kit; OSANG Healthcare, Anyang, Republic of Korea) and requiring oxygen were enrolled. All patients were managed according to existing guidelines.

### 2.2. Collected Data

Collected patient data included demographics, comorbidities, laboratory tests, and a dichotomous treatment outcome: hospital discharge or death. Laboratory tests were taken on admission day and included an automatic differential blood count and serum CRP concentration. The laboratory reference ranges for the performed tests were as follows: CRP 0.0–0.8 mg/dL; WBC 4.0–10.0 × 10^9^/L; PLT 150–400 × 10^9^/L; lymphocytes 0.9–4.5 × 10^3^/µL; neutrophils 1.9–8.0 × 10^3^/µL; monocytes 0.16–1.00 × 10^3^/µL; eosinophils 0.05–0.50 × 10^3^/µL; basophils 0.0–0.2 × 10^3^/µL. NLR was calculated by dividing the absolute count of neutrophils [1/µL] by an absolute lymphocyte count [1/µL].

### 2.3. Statistical Analysis

The gathered data was statistically processed. Results are presented as mean with standard deviation (SD) and median with interquartile range (IQR). The Shapiro–Wilk test was used to check the compliance with normal distribution. Differences between normally distributed data were checked with a *t*-test; otherwise, with the Mann–Whitney test. Univariable and multivariable logistic regression analyses were used to investigate the association with the mortality risk. Odds ratios were normalized by rounding to the nearest decimal place and multiplying the calculated values by 10. The ROC analysis was performed to determine the best predictive cut-off values using the Youden index. Based on the achieved results, a mortality prediction tool was built. A two-sided *p* < 0.05 was considered statistically significant.

### 2.4. Ethics

The study was approved by the Bioethics Committee of the Military Institute of Medicine (24/WIM/2021) on 21 May 2021 and conducted in accordance with the Declaration of Helsinki. All variables that could possibly jeopardize patients’ anonymity were permanently deleted from the dataset.

## 3. Results

The studied population comprised 306 patients (133 female gender, 173 male gender), aged 66.3 ± 15.2 years, of whom 105 (34.3%) died. The analysis of medical records showed that the study group included 175 (57.2%) patients with hypertension, 72 (25.3%) with diabetes, 65 (21.2%) patients with obesity, 42 (13.7%) with a history of cancer, 45 (14.7%) with chronic atrial fibrillation, 39 (12.7%) with chronic kidney disease, 32 (10.5%) with heart failure and 15 (4.9%) with chronic obstructive pulmonary disease. Moreover, 13 (4.2%) patients had a history of COVID-19 vaccination, and 24 (7.8%) were hospitalized in the ICU from the very beginning. In all patients, measurements of CBC and serum CRP concentration were performed. The results of the comparative analysis between survivors and deceased are presented in Table 1. In comparison to survivors, in the deceased group, lymphocyte and eosinophile counts were significantly lower—lymphocyte mean 0.83 × 10^3^/µL vs. 1.36 × 10^3^/µL, *p* = 0.002; eosinophile mean 0.03 × 10^3^/µL vs. 0.09 × 10^3^/µL, *p* = 0.009. In contrast, total white blood cells, neutrophils, serum CRP, and calculated NLR were substantially higher in those who died: 8.71 × 10^9^/L vs. 12.01 × 10^9^/L, *p* < 0.001; 6.62 × 10^3^/µL vs. 10.49 × 10^3^/µL, *p* < 0.001; 9.33 mg/dL vs. 14.59 mg/dL, *p* < 0.001; 9.19 vs. 33.62, *p* < 0.001, respectively.

The ROC analysis included different subpopulations of white blood cells that significantly differed between the considered groups, as well as calculated NLR and serum CRP. All parameters significantly distinguished survivors from deceased patients. Neutrophil count and NLR had the highest area under the curve (AUC), thus have better differentiating properties, especially in comparison to eosinophils (*p* = 0.027, *p* = 0.009; accordingly) and lymphocytes (*p* = 0.037, *p* < 0.001, accordingly). Moreover, the AUC of CRP concentration was not substantially different than NLR (*p* = 0.293) and neutrophils (*p* = 0.408). The best predictive cut-off values for mortality are presented in Table 2.

As neutrophils, lymphocytes, and eosinophils differed significantly between survivors and deceased, and all these variables substantially confirmed their usefulness for distinguishing survivors from deceased in ROC analysis, we decided to combine them into a single differentiating tool. Variables were converted from continuous to dichotomic—lower or higher than the cutoff value. Based on this, the Simplified White Blood Cell Score (WBCS) was developed. WBCS included neutrophils > 7.39 × 10^3^/µL, lymphocytes < 0.67 × 10^3^/µL, and eosinophils < 0.01 × 10^3^/µL coded as one or 0. Thus, WBCS ranges from 0 to three. In multivariable logistic regression analysis, all dichotomous WBCS parameters were significantly associated with mortality (Table 3).

**Figure 1:**
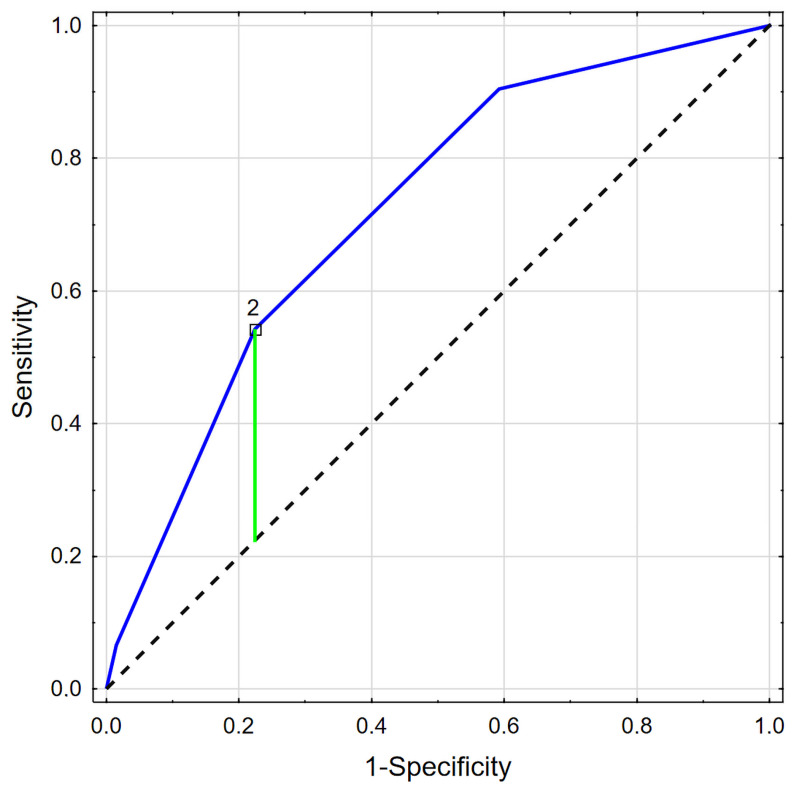
ROC analysis for WBCS and risk of death.

The value of WBCS was significantly related to mortality risk (OR = 2.821; CI: 2.037–3.906; *p* < 0.001). ROC analysis revealed that the best cut-off value for WBCS and mortality prediction is two (AUC 0.719; *p* < 0.001), as shown in Table 4 and Figure 1.

**Figure 2:**
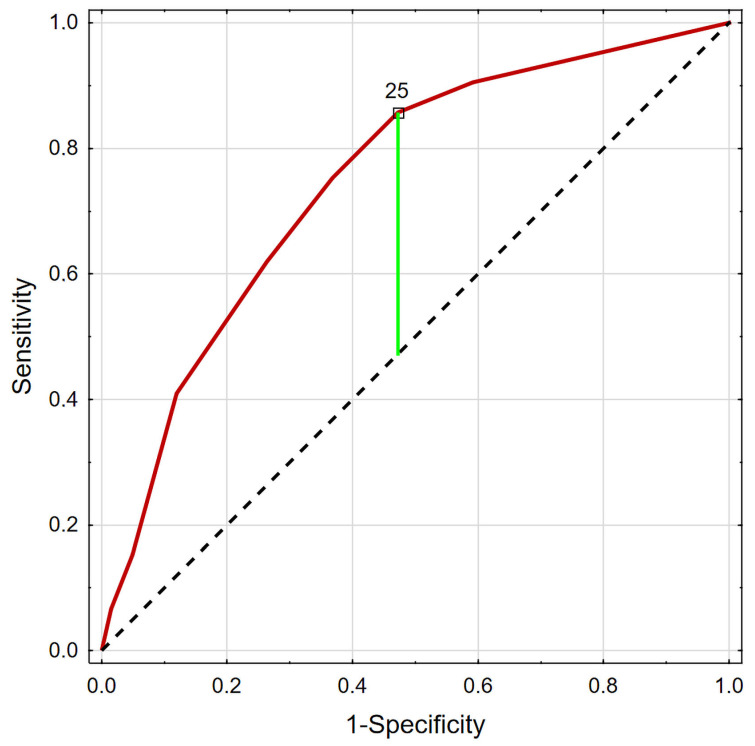
ROC analysis for modified WBCS and risk of death.

To improve the predictive properties of the WBCS, dichotomous values for each component variable were multiplied by the corresponding normalized odds ratios (25 for eosinophils, 20 for lymphocytes, and 55 for neutrophils) (Table 3). The modified WBCS showed a slight improvement in AUC (0.748; *p* < 0.001), but this somewhat complicated its use (Table 5). The proposed best cut-off value for mortality prediction was 25 (Figure 2).

In the backward multivariable logistic regression analysis, including variables significantly associated with mortality (demographics, comorbidities, inflammatory markers), WBCS and modified WBCS were independently associated with this outcome (Table 6).

## 4. Discussion

This study’s findings showed statistically significant differences between survivors and deceased in the population of patients with SARS-CoV-2 pneumonia. CRP concentration and calculated parameters—WBCS, NLR, taken on admission—were significantly associated with mortality. What is more, the neutrophil count and NLR had the largest AUC in ROC analysis, which makes them the best prognostic factors. According to study results, counts of neutrophils, lymphocytes, and eosinophils also significantly differ between populations of survivors and deceased. We evaluated the cut-off values to determine the significant threshold of respective values and then used them to create the WBCS. WBCS is a tool based on the three parameters calculated from the differential blood count. It is helpful in anticipating COVID-19 outcomes when used on admission. Undoubtedly, it is useful in very harsh circumstances—lack of resources, staff, and ICU beds. This study shows that the identification of high-risk patients who will need intensive treatment could be easily and quickly achieved on admission day. What is important, early identification of individuals at risk of death may facilitate early optimization of treatment strategy [11].

In ROC analysis, CRP concentration had the 3rd highest AUC, which makes CRP concentration a beneficial marker for predicting an unfavorable COVID-19 course. Parameters with higher AUC are NLR and neutrophils only. Nevertheless, mortality prediction derived only from serum CRP concentration can be misleading because it can be easily interfered with by steroids and non-steroid anti-inflammatory drugs intake [9,18,19,20].

Evaluating three different types of white blood cells—neutrophils, eosinophils, and lymphocytes—constitutes the usefulness of the WBCS. Different absolute counts of these types of immune cells were observed, depending on the type of infection—whether it was viral, fungal, or bacterial. Moreover, each of these three types of leukocytes was the subject of studies to anticipate a severe or fatal course of SARS-CoV-2 infection [21,22,23,24]

Although eosinophil count was not significantly associated with mortality prediction (OR 0.081; *p* = 0.177), we found a substantial difference in eosinophil count between survivors and deceased (*p* = 0.007). Eosinophils are a type of white blood cell that are frequently linked with allergic reactions and parasite infections; they are also a very useful marker to evaluate the course of the infection. Moreover, present studies connect eosinophils with contributions in such areas as modulation of immune response, regulation of homeostasis, tissue regeneration, glucose metabolism in adipocytes, autoimmunity, and defense against cancer and both viral and bacterial infection [25]. Changes in the absolute count of eosinophils correlate with the severity of the infection. In case of SARS-CoV-2 infection, there is evidence that a decrease in absolute eosinophil count is connected with a higher mortality rate [26]. What is more, a higher count of eosinophils is correlated with immune recovery and mild course of the disease, also with a better outcome [25,27]. It is worth mentioning that most of the COVID-19 deceased patients had eosinopenia, which was a significantly less frequent condition in patients who survived, both moderate and severe courses of the disease [28,29,30]. Study showed that patients with COVID-19 with eosinophilia had significantly lower serum CRP concentration and fewer ground-glass opacities in chest radiograms compared to patients without eosinophilia (*p* < 0.05) [27]. Also, patients with eosinophilia required shorter hospitalization, less often needed ICU admission, mechanical ventilation, and oxygen therapy [27]. Worth mentioning is the relation between viral load and absolute eosinophil count in infections. Studies have shown that the viral load of respiratory syncytial virus [28,31,32,33], parainfluenza virus [28,34], and rhinovirus [28,35] has a strong negative correlation with the level of eosinophils. In the influenza A virus infection, a higher level of eosinophils is connected with a faster removal of the virus [28,36,37,38]. Several studies suggested that allergic asthma coexisting with viral infection may be considered as a protective factor, compared with non-allergic asthma [25,39,40]. In fact, eosinophilia can be protective against SARS-CoV-2, even if resulting from allergic asthma, which corroborates our findings [25]. Other studies also showed that eosinophil count was increased in all patients before discharge, which implies that the growth of eosinophil level is correlated with improvement of clinical status in COVID-19 [23,29,30]. Conversely, another study showed that previous eosinophilia (≥0.15 × 10^9^/L) can cause lower hospital admission risk in patients with COVID-19 [25]. Moreover, in this work, patients with asthma and eosinophilia had lower mortality than patients with an eosinophil count below 0.15 × 10^9^/L. Furthermore, the protective role of eosinophils during SARS-CoV-2 and other viral infections is expressed even if an increase in absolute eosinophil count is caused by exacerbation of asthma [25,28,36,41,42].

Lymphocyte count is, according to this study, an important factor in predicting the mortal course of COVID-19. It is easy to evaluate and, what is more, it is cheap. Although the lymphocyte count has an AUC lower than NLR, neutrophil count, leukocyte count, and CRP concentration, it is still a useful tool. Its reliability is still on a high level, and therefore, it constitutes a beneficial addition to the WBCS. Studies showed that lymphopenia is also common among patients with COVID-19 [43,44,45]. On the other hand, the eosinophil level shows a positive correlation with the lymphocyte level, both in mild and severe courses of the disease. Another study suggests that lymphopenia could be an early accessible prognostic factor to determine the severity of the COVID-19 course in hospitalized patients [22]. Meta-data gathered from 76 studies from 16 countries indicates that lymphocyte count is the strongest factor, amongst 13 common laboratory variables that were investigated, to determine severe course and anticipate mortality [46]. Strong correlation was also found between lymphopenia and inflammatory biomarkers of COVID-19 [47]. Significant correlation was also found between lymphopenia and worsening of the radiological image [47]. Studies also showed that lymphopenia is a prognostic indicator of prolonged hospitalization [48,49]. Changes in absolute lymphocyte count can be a valuable marker not only in the COVID-19 course. For instance, lymphocyte level was significantly lower in patients with measles virus infection than in those without such infection [50]. Moreover, lymphopenia was also observed during Ebola, Marburg, and RSV infection [51,52]. It is suggested that the decrease in the lymphocyte level during these infections is a result of apoptosis [51,52]. Connection between lymphopenia, T lymphocyte subsets depletion, and SARS (Severe Acute Respiratory Syndrome) activity was also proved [53].

Findings of our study showed a significant correlation between a high level of absolute neutrophil count and mortality in a group of COVID-19 patients. What is more, absolute neutrophil count—and also NLR—has the largest AUC in ROC analysis. In that case, we can consider these two markers as strong and reliable factors of poor prognosis in the COVID-19 course. Absolute neutrophil count is significantly higher in patients with a severe course of COVID-19, similar to patients with SARS and Middle East respiratory syndrome (MERS). The shift toward immature neutrophils is a hyperinflammation marker, linked with exacerbation of the COVID-19 course [21]. Neutrophil count in the airway shows a positive correlation with the virulence and dose of the influenza virus. Studies showed that RSV can elongate a lifespan of neutrophils through inhibition or stopping the apoptosis. Additionally, during influenza A virus (IAV) infection in mice, IL-6 and granulocyte-colony stimulating factor (G-CSF) can elongate the lifespan of lung neutrophils. On the other hand, there are studies that imply that some viral infections can induce apoptosis in the neutrophil population, e.g., IAV or HIV. What is more, absolute neutrophil count rises during COVID-19, and it shows a positive correlation with the severity of the course. Worth mentioning is that in exceptionally severe cases and in poor prognosis, both neutrophil count and neutrophil extracellular traps (NET) had significantly increased levels. It was also reported that there is a connection between excessive activation of the anaphylatoxin–NET axis and thrombosis, and progression of the disease course in patients with COVID-19 [54]. It is also suggested that viral infection could inhibit neutrophil response and, because of that, make the system susceptible to bacterial or fungal infection [55]. What is more, bacterial and fungal coinfections involving existing viral respiratory tract infections are not uncommon. Both bacteria and fungi can be part of the usual commensal flora and also cause infections [56,57]. A good example is Candida albicans, which is present in the oral cavity as a part of the natural flora and can cause infections in cases of a weakened or overloaded immune system [58]. Patients with already developed infections (such as COVID-19) are particularly susceptible to this, but it is additionally facilitated by the use of broad-spectrum antibiotics and prolonged corticosteroid therapy [56,59]. The relevant factor is also the connection between some bacterial infections in COVID-19-positive patients and exacerbation of non-infectious diseases. For example, there is a strong correlation between chronic coronary syndrome and the presence of Staphylococcus aureus or a strong direct relationship between Hemophilus influenzae and pulmonary thromboembolism [56].

NLR is not only associated with high mortality risk in the COVID-19 course but also is known as a biomarker of increased severity of the disease; key clinical outcomes include intubation (more days intubated), ICU admission (longer ICU admission), and risk of severe disease in intubated patients [29,43,60,61,62,63,64,65,66,67]. Therefore, there are several fields in which calculating NLR can be very useful in the decision-making process, possibly even during triage procedures. An interesting and noteworthy fact about NLR is that it could be a prediction biomarker of influenza susceptibility, although it is not a prediction marker of a poor influenza course [68]. Amongst SARS-CoV-2, RSV, and influenza virus, only in SARS-CoV-2 infection is NLR a predictive factor of an unfavorable course of disease [69].

The usefulness of NLR in other viral diseases is an unexplored field, and it requires more studies to prove whether there is a proper way to use it.

On the other hand, it is worth mentioning that there were, in fact, many different approaches to anticipate a severe course of COVID-19. These methods include, e.g., assessment of peripheral and organ perfusion (fingertip infrared thermography, capillary refill time, dynamic tissue perfusion measurement, and pulse oximetry among others), diagnostic imaging (high-resolution computed tomography), hemostasis laboratory tests (elevated d-dimers and fibrinogen, prolonged prothrombin time and thrombocytopenia) and indexes derived from respiration parameters (OI—oxygenation index, OSI—oxygen saturation index, AOI—age-adjusted oxygenation index and ROX index—respiratory rate–oxygenation index) [70,71,72,73].

Comparing this work to other studies, there are several conclusions. According to Gajedra’s study, lymphopenia is the most common laboratory finding in patients with COVID-19 [14]. This is consistent with our research, which calculates the cut-off value for lower lymphocyte count to predict unfavorable severe COVID-19 outcomes.

As it was presented in this study, the cut-off value of NLR for mortality prediction in the COVID-19 course was 10.1 (sensitivity 0.629, specificity 0.711, AUC 0.715, *p* < 0.001), which significantly differs from Rathod’s et al. study, in which cut-off value for NLR was 4.14 (sensitivity 0.963, specificity 0.883, AUC 0.959, *p* < 0.001) [3]. These differences could be explained by variations in the population sample, duration of the study, or not including the asymptomatic patients in our study. On the other hand, according to Yan et al., NLR above the level of 11.75 was significantly correlated with all-cause in-hospital mortality [60]. Still, in Sejópoles et al.’s study, this cut-off value at admission was set at 6.13 [11]. Therefore, further studies or even meta-analysis would help proceed with the discussion about these cut-off values for NLR.

Similarly to NLR, initial CRP concentration cut-off values anticipating the severe course of COVID-19 and high mortality risk differ in other studies. Stringer et al. set this cut-off value at 4.0 mg/dL, but Smilowitz et al. suggested the CRP concentration threshold of 10.8 mg/dL [19,74]. The findings of our study showed that the cut-off value at 6.9 mg/dL should also be considered.

In another study, Xuan et al. investigated 99 patients with COVID-19 treated in the ICU, 58 of whom survived, and 41 died. Low absolute eosinophil count was a prognostic indicator of fatal course due to COVID-19 with a cut-off eosinophil value of 0.04 × 10^9^/L [75]. Therefore, eosinophils were included in our ROC analysis and WBCS calculation. However, in our study, the calculated eosinophil count cut-off value was 0.01 × 10^9^/L, and WBCS can be stricter than in the Xuan et al. study.

Without a doubt, there are many reasons why some of the findings of our study differ from those of other studies. Gathering data from all around the world can lead to similar but slightly different conclusions. One of the most important factors is time—it can indicate which variant or strain could provoke a new wave of coronavirus disease, and different variants may vary in the characteristics of infected patients. Another important factor is the demography of patients in the collected data. Some of the healthcare units could prioritize a specific group of patients, depending on their specialty. This situation can refer to a population from a specific area, with a specific kind of disease, or to people with a specified profession. Each group of patients has its own attributes, which we should pay attention to.

Nevertheless, depending on the different sample, study inclusion/exclusion criteria, population immunity, geographical localization, or even different diagnostic methods, the optimal cut-off value of specific parameter concentration may vary. These differences prove that there is a need for further studies and validations [19,74].

Despite global efforts, SARS-CoV-2 has not been eliminated. Although the number of cases and deaths of COVID-19 is decreasing, which is caused by, i.e., vaccinations, new variants of concern are being discovered; thus, it is reasonable to maintain useful tools and methods in the future [76,77].

This study has several limitations. It is a single-center retrospective study based on the findings from hospitalizations during the domination of one variant of SARS-CoV-2—the Delta variant. Further validations are advised for the other variants of concern, such as the Omicron variant, which is a dominant variant nowadays. Although the Omicron variant is considered more benign than the Delta variant (less risk of hospitalization, ICU admission, oxygen therapy, mechanical ventilation, and death), it should not be disregarded, as it still can lead to a severe course of COVID-19 [78,79]. One of the significant differences between these variants is a less pronounced loss of smell in the Omicron variant, possibly due to reduced viral load in nasal tissue [78,79]. This could lead to delayed onset of treatment or possible misdiagnosis (without laboratory tests). What is more, this study took place when the vaccination level was not high. This leads to the conclusion that nowadays, the immune response could be different, and we need more data. The next limitation is the fact that this study did not include children and pregnant women, and it narrows the study population to non-pregnant European adults. That means we do not have enough supporting data to easily transfer the usefulness of WBCS to the mentioned groups. Our findings could be applicable; however, strong validation is needed. Another limitation is the lack of a control group and the exclusion of asymptomatic patients.

Authors recommend further studies to assess the usefulness of WBCS in COVID-19 patients and in other viral infections, caused by, e.g., the influenza virus or syncytial respiratory virus. Additionally, validation in non-European centers is needed.

## 5. Conclusions

Differential blood count-related variables, especially counts of neutrophils, lymphocytes, eosinophils, and NLR, can be helpful in identifying patients with a high risk of death due to the unfavorable course of severe COVID-19 infection. However, the use of all these parameters separately could be cumbersome in clinical practice. The proposed new simplified White Blood Count Score is easy to use and has equal or even better mortality prediction properties compared to common differential blood count-related variables and C-reactive protein. However, further studies are needed to validate this tool in different patient populations.

## Figures and Tables

**Table 1:** Comparison of CBC and serum CRP between survivors and deceased.

	All Patients (*n* = 306)		Survivors (*n* = 201)		Deceased (*n* = 105)		Significance
	Median (Mean)	IQR (±SD)	Median (Mean)	IQR (±SD)	Median (Mean)	IQR (±SD)	*p*-Value
RBC (×10^12^/L)	4.32 (4.24)	1.03 (0.71)	4.37 (4.27)	1.05 (0.71)	4.26 (4.17)	0.90 (0.69)	0.007
HGB (g/dL)	12.90 (12.71)	3.20 (2.21)	13.10 (12.75)	3.10 (2.24)	12.70 (12.63)	3.30 (2.17)	0.001
PLT (×10^9^/L)	207 (223.79)	118 (109.29)	210 (224.68)	115 (109.92)	203 (222.10)	116 (108.58)	0.611
WBC (×10^9^/L)	7.79 (9.84)	5.23 (8.60)	6.97 (8.71)	4.08 (8.38)	9.29 (12.01)	6.25 (8.65)	<0.001
Lymphocytes (×10^3^/µL)	0.78 (1.18)	0.58 (4.27)	0.82 (1.36)	0.58 (5.25)	0.66 (0.83)	0.55 (0.62)	0.002
Neutrophils (×10^3^/µL)	6.40 (7.95)	5.36 (6.23)	5.35 (6.62)	3.92 (5.02)	8.67 (10.49)	6.23 (7.44)	<0.001
Monocytes (×10^3^/µL)	0.36 (0.43)	0.28 (0.39)	0.36 (0.42)	0.28 (0.41)	0.36 (0.45)	0.32 (0.35)	0.445
Basophils (×10^3^/µL)	0.03 (0.06)	0.04 (0.12)	0.03 (0.05)	0.04 (0.10)	0.03 (0.08)	0.04 (0.16)	0.233
Eosinophils (×10^3^/µL)	0.01 (0.07)	0.03 (0.40)	0.01 (0.09)	0.03 (0.49)	0.01 (0.03)	0.02 (0.06)	0.009
NLR	8.14 (17.57)	10.11 (74.61)	6.05 (9.19)	7.23 (10.40)	12.73 (33.62)	15.25 (125.39)	<0.001
CRP (mg/dL)	9.65 (11.12)	12.25 (8.40)	7.75 (9.33)	10.80 (7.37)	12.50 (14.59)	11.50 (9.19)	<0.001

RBC, red blood cell concentration; HGB, hemoglobin concentration; PLT, platelet concentration; WBC, white blood cell concentration.

**Table 2:** Results of ROC analysis of the considered variables.

	Cut-Off	Sensitivity	Specificity	AUC	Significance—*p*
WBC (×10^9^/L)	7.94	0.667	0.632	0.668	<0.001
RBC (×10^12^/L)	4.51	0.705	0. 428	0.554	0.114
HGB (g/dL)	13.4	0.657	0.433	0.530	0.379
Lymphocytes (×10^3^/µL)	0.67	0.514	0.687	0.608	0.002
Neutrophils (×10^3^/µL)	7.39	0.629	0.736	0.707	<0.001
Eosinophils (×10^3^/µL)	0.01	0.686	0.468	0.591	0.008
NLR	10.1	0.629	0.711	0.715	<0.001
CRP (mg/dL)	6.9	0.843	0.444	0.681	<0.001

**Table 3:** Results of multivariable regression analysis of investigated variables for the prediction of mortality risk.

	OR	CI (−95%; +95%)	Significance—*p*
Lymphocytes	1.973	1.165; 3.341	0.011
Neutrophils	5.477	3.163; 9.484	<0.001
Eosinophils	2.524	1.415; 4.503	0.002

CI—confidence interval; OR—odds ratio.

**Table 4:** Results of ROC analysis of WBCS compared to deceased patients.

WBCS	Sensitivity (%)	Specificity (%)	Cumulative Deaths N (%)
0	100.0	0.0	10/92 (10.9)
1	90.5	40.8	48/204 (23.5)
2	54.3	77.6	98/296 (33.1)
3	6.7	98.5	105/306 (34.1)

**Table 5:** Results of ROC analysis of modified WBCS compared to deceased patients.

Modified WBCS	Sensitivity (%)	Specificity (%)	Cumulative Deaths N(%)
0	100.0	0.0	10/92 (10.9)
20	90.5	40.8	15/121 (12.4)
25	85.7	52.7	26/153 (17.0)
45	75.2	63.2	40/188 (21.3)
55	61.9	73.6	62/239 (25.9)
75	41.0	88.1	89/280 (31.8)
80	15.2	91.0	98/296 (33.1)
100	6.7	98.5	105/306 (34.3)

**Table 6:** Results of logistic regression analysis.

Variable	OR	CI (−95%; +95%)	Significance—*p*
Univariable analysis			
**Age**	1.045	1.026; 1.065	<0.001
**Gender M**	0.840	0.541;1.399	0.566
**ICU first**	2.066	0.893; 4.778	0.090
**Obesity**	1.258	0.713; 2.218	0.428
**Neoplasm history**	1.360	0.698; 2.650	0.366
**Hypertension**	1.948	1.189; 3.191	0.008
**CKD**	3.243	1.628; 6.460	<0.001
**Heart failure**	1.354	0.640; 2.862	0.428
**CAF**	1.194	0.620; 2.298	0.596
**Diabetes mellitus**	3.008	1.746; 5.183	<0.001
**COPD**	2.286	0.805; 6.488	0.120
**Vaccination status**	1.719	0.502; 5.886	0.388
**CRP**	1.080	1.047; 1.114	<0.001
**NLR**	1.054	1.028; 1.080	<0.001
**WBCS**	2.821	2.037; 3.906	<0.001
**WBCS modified**	1.032	1.023; 1.042	<0.001
Backward multivariable analysis (WBCS model)			
**Age**	1.045	1.022; 1.068	<0.001
**Diabetes mellitus**	3.137	1.664; 5.916	<0.001
**CRP**	1.077	1.039; 1.117	<0.001
**WBCS**	2.319	1.623; 3.312	<0.001
Backward multivariable analysis (modified WBCS model)			
**Age**	1.044	1.022; 1.068	<0.001
**Diabetes mellitus**	2.795	1.476; 5.293	0.002
**CRP**	1.073	1.035; 1.113	<0.001
**WBCS modified**	1.025	1.015; 1.035	<0.001

CAF—chronic atrial fibrillation; CKD—chronic kidney disease; CRP—C-reactive protein; COPD—chronic obstructive pulmonary disease; ICU—intensive care unit; NLR—neutrophil to lymphocyte ratio; WBCS—White Blood Cell Score.

## Data Availability

The datasets used and analyzed in this study are available from the corresponding author upon reasonable request.

## Abbreviations

The following abbreviations are used in this manuscript:

SARS-CoV-2	severe acute respiratory syndrome coronavirus 2
WBCS	White Blood Cell Score
SIRS	systemic inflammatory response syndrome
WHO	World Health Organization
MODS	multiple organ dysfunction syndrome
NLR	neutrophil to lymphocyte ratio
CRP	C-reactive protein
LDH	lactate dehydrogenase
CBC	complete blood count
CAP	community-acquired pneumonia
G-CSF	granulocyte colony-stimulating factor
HIV	human immunodeficiency virus
WBC	white blood cell concentration
RBC	red blood cell concentration
HGB	hemoglobin concentration
PLT	platelet concentration
AUC	area under the curve
OR	odds ratio
CI	confidence interval
ICU	intensive care unit
RSV	respiratory syncytial virus
SARS	severe acute respiratory syndrome
MERS	Middle East respiratory syndrome
IAV	influenza A virus
IL-6	interleukin 6
NET	neutrophil extracellular traps
